# Association of temperature and precipitation with malaria incidence in 57 countries and territories from 2000 to 2019: A worldwide observational study

**DOI:** 10.7189/jogh.14.04021

**Published:** 2024-02-23

**Authors:** Qiao Liu, Yaping Wang, Jie Deng, Wenxin Yan, Chenyuan Qin, Min Du, Min Liu, Jue Liu

**Affiliations:** 1 Department of Epidemiology and Biostatistics, School of Public Health, Peking University, Beijing, China; 2 Key Laboratory of Epidemiology of Major Diseases (Peking University), Ministry of Education, Haidian District, Beijing, China; 3 Institute for Global Health and Development, Peking University, Haidian District, Beijing, China

## Abstract

**Background:**

The transmission of malaria is known to be affected by climatic factors. However, existing studies on the impact of temperature and precipitation on malaria incidence offer no clear-cut conclusions, and there is a lack of research on a global scale. We aimed to estimate the association of temperature and precipitation with malaria incidence globally from 2000 to 2019.

**Methods:**

We used meteorological data from the National Centers for Environmental Information and malaria incidence data from the Global Burden of Disease Study 2019 to calculate effect sizes through quasi-Poisson generalised linear models while controlling for confounders.

**Results:**

231.4 million malaria cases occurred worldwide in 2019. National annual average temperature and precipitation were associated with malaria incidence, with an increase in the age-standardised incidence rate (ASIR) of 2.01% (95% confidence interval (CI) = 2.00, 2.02) and 6.04% (95% CI = 6.00, 6.09) following one unit increase of national annual average temperature and precipitation. In subgroup analysis, we found that malaria incidence in Asian countries was most affected by temperature, while the incidence in African countries was most affected by precipitation (*P* < 0.05). Stratified by age, children under five were most affected by both temperature and precipitation (*P* < 0.05). We additionally found that the impact of the national annual average temperature on malaria incidence increased over time (*P* < 0.05).

**Conclusions:**

We advocate for a comprehensive approach to malaria prevention, focussed on addressing the impact of climate factors through international collaboration, adaptive measures, and targeted interventions for vulnerable populations.

Malaria remains a major public health challenge which impacts people’s health and livelihoods worldwide, despite being preventable and curable. According to the World Health Organization (WHO), an estimated 247 million malaria cases occurred in 84 malaria-endemic countries in 2021 compared to 245 million in 2020, with most of this increase coming from African countries [1]. Although these African countries accounted for approximately 95% of global malaria cases in 2021, it is essential to acknowledge the burden in other world regions. Countries in Southeast Asia, for instance, contributed to about 2% of the global malaria cases [1].

There were an estimated 230 million malaria cases globally in 2015; this became the baseline year of the Global Technical Strategy for Malaria 2016–2030 (GTS), which set its first milestone in 2020 to reduce malaria incidence and mortality by at least 40%. However, the progress has stalled or reversed globally [2,3]. A previous observational study also raised concerns, indicating that in 2019, 40 countries exhibited higher age-standardised incidence rates (ASIRs) of malaria compared to 2015 [4], especially in sub-Saharan African countries, followed by South America and South Asia. Various factors may account for this stagnation or regression, including the escalation of artemisinin resistance and disruptions to health systems caused by the ongoing challenges posed by the coronavirus disease 2019 (COVID-19) pandemic [5,6]. For example, the pandemic indirectly caused an increase in the prevalence of malaria, as well as an estimated additional 13.4 million cases due to health care service disruptions between 2019 and 2021, hindering progress and exacerbating the challenges in malaria control [1,7].

Malaria is caused by a parasite that is transmitted to the human host by the *Anopheles* genus of mosquitoes. The survival and longevity of these mosquitoes, as well as the rate of multiplication of the parasite within them, are influenced by climatic factors, especially temperature [8]. Consequently, temperature, rainfall, humidity, wind, and daylight duration all affect malaria transmission. For example, increasing temperature could result in the extension of the windows of malaria transmission, the temporal distribution, and the man-hour density of *Anopheles Culicifacies* and *Anopheles Fluviatilis* [9]. A study in China, which used a distributed lag nonlinear model to quantify the effect of different temperature measures on malaria, found that each 5°C increase in average temperature above 10°C was associated with a 22% (95% confidence interval (CI) = 17, 28) increase in malaria cases [10]. A systematic review predicted that further increasing temperatures would extend the seasonality of malaria transmission, enabling it to occur for up to six months annually in 2051–80 in European countries [11]. A modelling study showed that increased temperature variations were related to higher probabilities of *Aedes* species’ occurrence, and the optimal occurrence would take place when the temperature variations from the mean were above 25°C [12]. Conversely, a previous observational study found a negative relationship between temperature and malaria incidence rate, with a sharp decrease in incidence rate during 2000–03 and 2012–14, respectively, occurring with increasing average temperatures in Ghana and Nigeria [13].

The relationship between precipitation and malaria transmission is intricate. On one hand, precipitation-induced standing water provides a breeding habitat for mosquitoes; [14] on the other hand, high-intensity rainfall may wash away or kill both larvae and adult mosquitoes in some cases [12]. A study reported a positive correlation between rainfall and malaria prevalence in Thailand [15]. Another modelling study found that the probability of *Aedes* species’ occurrence increased to nearly 99% when precipitation was less than 100 mm during the warmest quarter of the year, but this probability quickly dropped as precipitation exceeded 300 mm, reaching 30% [12]. This complex relationship suggests that precipitation plays a dual role in promoting mosquito breeding and malaria transmission while, under extreme conditions, it may have adverse effects.

In the context of global climate change, malaria prevention and control efforts are facing greater challenges. The existing literature on the impact of temperature and precipitation on malaria incidence is inconclusive, and global-scale studies are scarce. Therefore, we aimed to estimate the association of national annual average temperature and precipitation with malaria incidence from 2000 to 2019. By analysing data spanning 20 years from countries worldwide, we sought to provide a comprehensive view of the global distribution of national annual average temperature and precipitation, as well as their association with malaria incidence. Our findings can complement previous studies and help improve the understanding of the impact of climate factors on malaria transmission, while also informing ongoing efforts to control malaria amidst climate change, thereby assisting in achieving the global goal of malaria elimination.

## METHODS

### Study design and data sources

This was a worldwide observational study that encompassed all the countries and territories that were malaria-endemic and reported daily temperature and precipitation from 2000 to 2019. We used meteorological data from the National Centers for Environmental Information (NCEI) [16], which operates under the National Oceanic and Atmospheric Administration and provides environmental data, products, and services. We retrieved malaria incidence data from the Global Burden of Disease Study 2019 (GBD 2019) [17], which explores and presents the comparative magnitude of health losses due to diseases by sex, age, and location over time [18]. We extracted data on socioeconomic and socio-demographic characteristics, and health resources from the open-source database of the World Bank [19] to use as covariates in the analyses.

### National annual average temperature and precipitation

We extracted daily temperature data and total precipitation data for a specific site on all dates within a year from the NCEI website. However, the original data did not always cover all 365 days, meaning some covered over 300 days, and some only a few dozen days. We merged and processed the daily temperature and total precipitation data of all meteorological observation stations involved each year and finally obtained the daily temperature and total precipitation data of global meteorological stations from 2000 to 2019.

### Malaria incidence data

We extracted annual cases and incidence rates of malaria from 2000 to 2019, stratified by sex, age, and country, from the Global Health Data Exchange, established by the GBD group. We also retrieved age-standardised incidence rates (ASIRs) of malaria, which were calculated by applying the age-specific rates to a GBD world standard population and were used to compare populations with different age structures or for the same population over time in which the age profiles change accordingly. Using this data, we performed sub-analyses by age groups (<5, 5–14, 15–49, 50–69, and ≥70 years). The general methodological approaches to estimate the incidence of malaria infection were described elsewhere [20]. Briefly, all available data on incidence were standardised and pooled into a single database, which was then used to generate cause-specific estimates by age, sex, year, and geography; afterwards, multiple models, including cause of death ensemble modelling, disease model-Bayesian meta-regression, comorbidity correction, and others to estimate comparable data of different diseases across the world [20].

### Covariates

We used data on socioeconomic and sociodemographic characteristics and health resources as covariates. We extracted the socio-demographic index (SDI) values from the Global Health Data Exchange. The SDI, developed by GBD researchers, is a composite indicator of the total fertility rate aged <25 years, years of education for those aged ≥15 years, and lag-distributed income per capita [21]. Current health expenditure per capita, percentage of population practising open defecation, carbon dioxide emissions (metric tons per capita) (CO_2_), particulate matter smaller than 2.5 µm (PM2.5) air pollution, mean annual exposure (micrograms per cubic meter), percentage of population using at least basic drinking water services, and percentage of population using at least basic sanitation services were all extracted from the World Bank.

### Statistical analysis

Based on the daily temperature and total precipitation values of all stations, we obtained the average daily temperature and total precipitation data for all days of each year to obtain the annual average temperature and precipitation data for all stations. Finally, based on the geographic location of each meteorological observation station, we calculated the annual average temperature and precipitation data of each country. We converted the units of temperature and precipitation into °C and mm.

In the multivariable regression analysis, we used the quasi-Poisson generalised linear model (GLM) [22] to calculate the effects of temperature and precipitation on malaria incidence after controlling for potential confounders and considering the overdispersion trend of data on malaria incidence. The quasi-Poisson GLM is the most common way to deal with overdispersion for counts; its variance is a linear function of the mean to which the weights are directly proportional [23]. Our choice of the quasi-Poisson model was driven by the nature of data, as malaria incidence is a discrete variable representing the number of cases, and is often characterised by overdispersion. The formula was as follows:

*log(y(t)) = α + β_1_AT_ij_ + β_2_AP_ij_ + β_3_SDI_ij _+ β_4_CHE_ij_ + β_5_POD_ij_ + β_6_CO2_ij_ + β_7_PM2.5_ij_ + β_8_PDW_ij_ + β_9_PBS_ij_*

where *Y*(*t*) refers to the expected number of new cases per 100 000 population on time *t*; α refers to the intercept; β refers to the regression coefficient; AT*_ij_* refers to the annual average temperature in the year *i* of country *j*; AP*_ij_* refers to the annual average precipitation in the year *i* of country *j*; SDI*_ij_* refers to SDI in the year *i* of country *j*; CHE*_ij_* refers to current health expenditure per capita in the year *i* of country *j*; POD*_ij_* refers to the percentage of population practising open defecation in the year *i* of country *j*; CO2*_ij_* refers to carbon dioxide emissions (metric tons per capita) in the year *i* of country *j*; PM2.5*_ij_* refers to PM2.5 air pollution, mean annual exposure (micrograms per cubic meter) in the year *i* of country *j*; PDW*_ij_* refers to the percentage of population using at least basic drinking water services in the year *i* of country *j*; and PBS*_ij_* refers to the percentage of population using at least basic sanitation services in the year *i* of country *j*.

We presented effect sizes as a percentage change (%) in the number of cases per 100 000 population caused by each 1 unit change in AT, AP or SDI, calculated using the formula (e*^β^* − 1) × 100%. Moreover, we controlled for HDI in the multivariable regression models instead of SDI to assess the stability of the results.

We performed all analyses using Microsoft Excel, version 16.78.3 (Microsoft Corporation, San Francisco, CA, USA), and *R*, version 4.0.4 (R Core Team, Vienna, Austria). All tests were two-sided, and a *P*-value <0.05 was considered statistically significant.

## RESULTS

### Global and national burden of malaria from 2000 to 2019

In 2000, there were 252.9 million malaria cases globally, with an ASIR of 3946.18 per 100 000 population. This increased to 263.7 million cases in 2010, while the ASIR of malaria decreased to 3877.86 per 100 000 population. By 2019, the number of malaria cases decreased to 231.4 million and the related ASIR decreased to 3247.02 per 100 000 population. Children <5 years old contributed the most to the malaria, accounting for 42.09%, 40.56%, and 36.82% of all malaria cases in 2000, 2010, and 2019, respectively.

**Figure 1:**
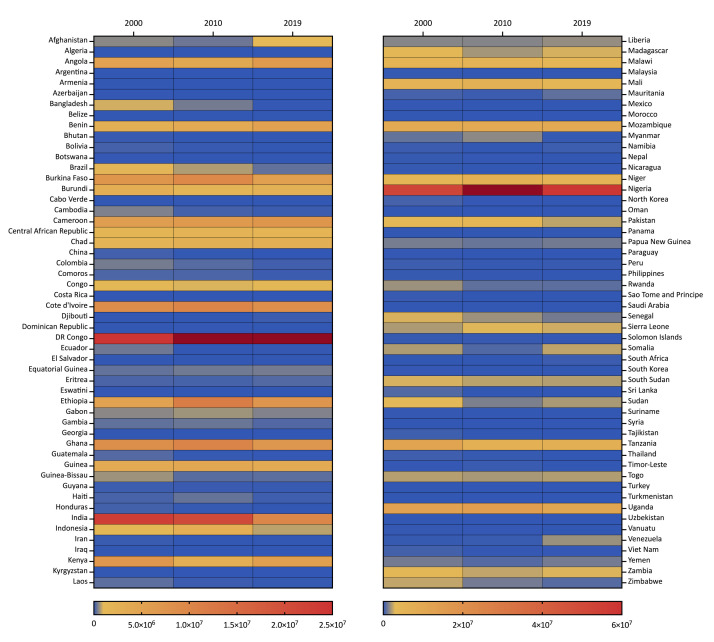
Heat map of malaria cases in different countries in 2000, 2010, and 2019.

Nigeria, DR Congo, and India had the highest number of malaria cases in 2000 (52.3, 24.5, and 22.9 million, respectively) and 2010 (64.0, 29.0, and 20.7 million, respectively). While Nigeria and DR Congo still had the greatest burden of cases in 2019 (58.1 and 27.7 million, respectively), India was overtaken by Uganda (10.7 million) (**Figure 1**).

**Figure 2:**
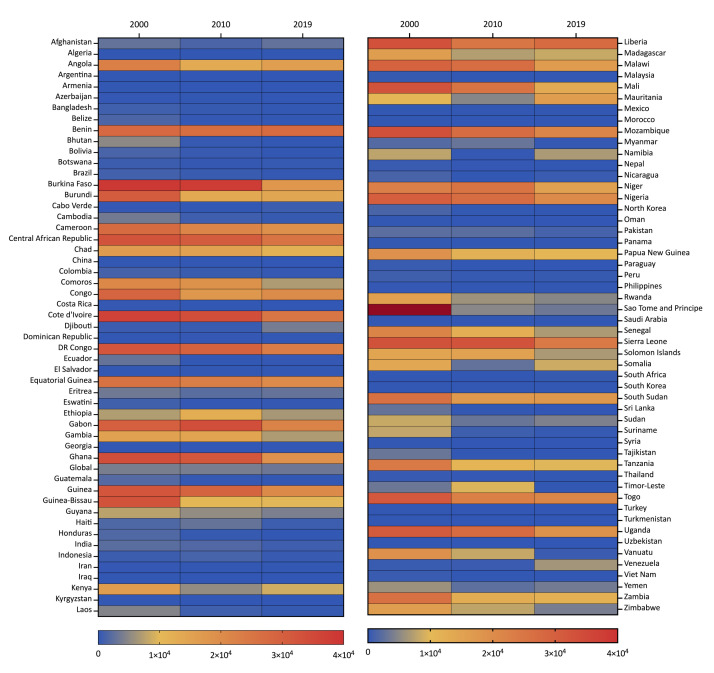
Heat map of malaria ASIR in different countries in 2000, 2010, and 2019.

Sao Tome and Principe had the highest ASIR of malaria in 2000 (44647.26 per 100 000 population), followed by Burkina Faso (38844.59 per 100 000 population) and Cote d’Ivoire (36450.25 per 100 000 population) (**Figure 2**). Meanwhile, Burkina Faso (37543.62 per 100 000 population) and Cote d’Ivoire (33748.09 per 100 000) had the highest ASIR in 2010, followed by Gabon (33570.31 per 100 000 population). In 2019, Benin had the highest ASIR of malaria (27623.45 per 100 000 population), followed by Liberia (27428.04 per 100 000 population) and the Central African Republic (25301.00 per 100 000 population).

### National annual average precipitation and temperature from 2000 to 2019

**Figure 3:**
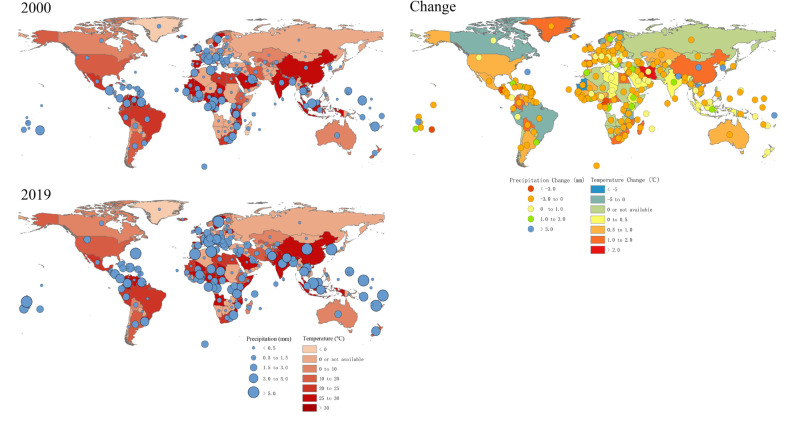
National annual average precipitation and temperature in 2000 and 2019, and their change between 2000 and 2019.

**Figure 4:**
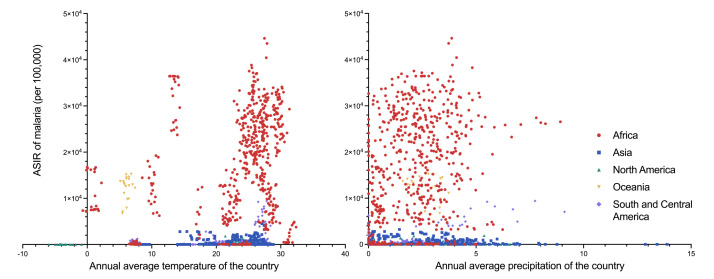
ASIR of malaria, with national annual average temperature or precipitation. ASIR – age-standardised incidence rate.

In 2000, the national annual average temperature of 69 countries was over 20.0°C, and was the highest in Nigeria (28.9°C), followed by Singapore (28.7°C) and Mali (28.6°C) (**Figure 3**). The national annual average temperature in Nigeria was even higher in 2019 (29.5°C). Besides Nigeria, 97 countries had higher national annual average temperature in 2019 than in 2000, with the maximum temperature increase in Nepal (from 18.4 to 21.7°C), followed by DR Congo (from 27.6 to 30.4°C) and Ecuador (from 18.4 to 20.7°C). In 2000, among the 21 countries with malaria ASIR over 10 000 per 100 000 population, 17 had a national annual average temperature >20.0°C, of which 13 had an average temperature >25.0°C. In 2019, among the 18 countries with malaria ASIR over 10 000 per 100 000 population, 17 had their national annual average temperature over 20.0°C, and a further 14 had a temperature >25.0°C (**Figures 3–4**).

As for national annual average precipitation, 31 countries had precipitation over 3.0 mm per day in 2000, with the highest in Micronesia (12.1 mm per day), followed by Palestine (11.4 mm per day) and Bahrain (8.0 mm per day). Fifty-eight countries had a higher national annual average precipitation in 2019 than in 2000, with the maximum precipitation increase in Tuvalu (from 2.8 to 10.1 mm per day), followed by China (from 1.3 to 7.3 mm per day) and South Korea (from 1.2 to 6.9 mm per day) (**Figure 3**). In 2000, among the 21 countries with malaria ASIR over 10 000 per 100 000 population, 12 had a national annual average precipitation >2.0 mm per day, and a further six had precipitation >3.0 mm per day. In 2019, 11 of 18 countries with malaria ASIR over 10 000 per 100 000 population had a national annual average precipitation >2.0 mm per day, and five had precipitation of >3.0 mm per day (**Figure 4**).

### Association of annual average temperature and precipitation with malaria incidence rates

We included 57 malaria-endemic countries with meteorological observation data in the multivariable regression analysis (**Table 1**).

**Table 1:** Estimated rate changes (per 100 000) with 95% CIs associated with each one unit increase in annual average temperature, annual average precipitation, and SDI among studied countries, 2000–19*

Group	Annual average temperature (%) (95% CI)	*P*-value	Annual average precipitation (%) (95% CI)	*P*-value	SDI (%) (95% CI)	*P*-value
All	2.01 (2.00, 2.02)	<0.001	6.04 (6.00, 6.09)		−0.78 (−0.79, −0.77)	
Location						
*Africa*	1.35 (1.34, 1.37)	<0.001	7.52 (7.48, 7.57)	<0.001	−1.37 (−1.38, −1.36)	<0.001
*Asia*	22.69 (22.36, 23.03)	<0.001	−8.38 (−8.75, −8.00)	<0.001	−2.64 (−2.78, −2.50)	<0.001
*North America*	8.49 (7.87, 9.13)	<0.001	5.47 (4.45, 6.49)	<0.001	11.63 (10.01, 13.28)	<0.001
*Oceania*	2.79 (1.87, 3.73)	<0.001	6.80 (5.20, 8.43)	<0.001	−11.96 (−15.35, −8.43)	<0.001
*South and Central America*	18.99 (18.67, 19.31)	<0.001	0.32 (0.00, 0.64)	<0.05	3.05 (2.79, 3.31)	<0.001
Sex						
*Male*	2.01 (2.00, 2.02)	<0.001	6.04 (6.00, 6.09)	<0.001	−0.79 (−0.79, −0.78)	<0.001
*Female*	2.01 (2.00, 2.02)	<0.001	6.05 (6.00, 6.09)	<0.001	−0.78 (−0.79, −0.77)	<0.001
Age						
*<5 y group*	2.76 (2.75, 2.77)	<0.001	8.07 (8.04, 8.09)	<0.001	−0.91 (−0.91, −0.90)	<0.001
*5–14 y group*	2.44 (2.43, 2.45)	<0.001	7.25 (7.22, 7.29)	<0.001	−0.97 (−0.98, −0.96)	<0.001
*15–49 y group*	1.36 (1.35, 1.38)	<0.001	4.32 (4.27, 4.37)	<0.001	−0.57 (−0.58, −0.55)	<0.001
*50 − 69 y group*	0. 92 (0.90, 0.94)	<0.001	2.13 (2.05, 2.21)	<0.001	−0.39 (−0.41, −0.38)	<0.001
*>70 y group*	0.21 (0.18, 0.23)	<0.001	−0.15 (−0.27, −0.04)	<0.01	−0.41 (−0.44, −0.39)	<0.001

CI – confidence interval, SDI – socio-demographic index

*The models were adjusted for SDI, Current health expenditure per capita, percentage of population practising open defecation, carbon dioxide emissions (metric tons per capita), PM2.5 air pollution, mean annual exposure (micrograms per cubic meter), percentage of population using at least basic drinking water services, and percentage of population using at least basic sanitation services. Countries included in the analysis: Afghanistan, Angola, Bangladesh, Bhutan, Bolivia, Brazil, Burkina Faso, Cabo Verde, Cameroon, Central African Republic, Chad, Colombia, Congo, Côte d’Ivoire, DR Congo, Djibouti, Dominican Republic, Ecuador, El Salvador, Equatorial Guinea, Eswatini, Ethiopia, Gabon, Gambia, Ghana, Guatemala, Guyana, India, Indonesia, Iran, Kenya, Laos, Madagascar, Malaysia, Mali, Mauritania, Mexico, Mozambique, Nepal, Nicaragua, Niger, Nigeria, Pakistan, Panama, Peru, South Korea, Rwanda, Sao Tome and Principe, Saudi Arabia, Senegal, Sierra Leone, Solomon Islands, South, Africa, Thailand, Uganda, Tanzania, and Venezuela.

The multivariable quasi-Poisson GLM regression models showed that, if the national annual average temperature were to increase by 1°C, the ASIR of malaria would increase by 2.01% (95% CI = 2.00, 2.02). According to the subgroup analyses, malaria incidence in Asian countries was most affected by temperature, with an increase in ASIR of 22.69% (95% CI = 22.36, 23.03) following a 1°C increase in the national annual average temperature. Despite the extremely high ASIR, African countries were least affected by temperature, with an increase in ASIR of 1.35% (95% CI = 1.34, 1.37) following a 1°C increase in national annual average temperature. As for different age groups, children <5 years were most affected by temperature, with an incidence rate of malaria increasing by 2.76% (95% CI = 2.75, 2.77) when the national annual average temperature increased by 1°C.

The models also showed that, if the national annual average precipitation were to increase by one mm per day, the ASIR of malaria would increase by 6.04% (95% CI = 6.00, 6.09). In the subgroup analyses, African countries were the most affected by precipitation, with an increase in malaria ASIR of 7.52% (95% CI = 7.48, 7.57) following a one mm increase in national annual average daily precipitation. However, in Asian countries, when the national annual average precipitation increases by one mm per day, the ASIR of malaria decreases by 8.38% (95% CI = 8.00, 8.75). Children under the age of five were also affected most by precipitation, with the incidence rate of malaria increasing by 8.07% (95% CI = 8.04, 8.09) when the national annual average precipitation increased by one mm per day.

### Association of SDI with malaria incidence rates

The multivariable quasi-Poisson GLM regression models predicted that an SDI increase of 0.01 led to a decrease of malaria ASIR by 0.78% (95% CI = 0.77, 0.79) (**Table 1**). In the subgroup analyses, malaria incidence rates in Africa, Asia, and Oceania were likely to decrease when SDI increased. However, in North American countries and South and Central American countries, the ASIR of malaria would increase by 11.63% (95% CI = 10.01, 13.28) and 3.05% (95% CI = 2.79, 3.31), respectively, with a 0.01 increase in the SDI.

### Trends of impact of temperature, precipitation and SDI on malaria incidence, from 2000 to 2019

**Figure 5:**
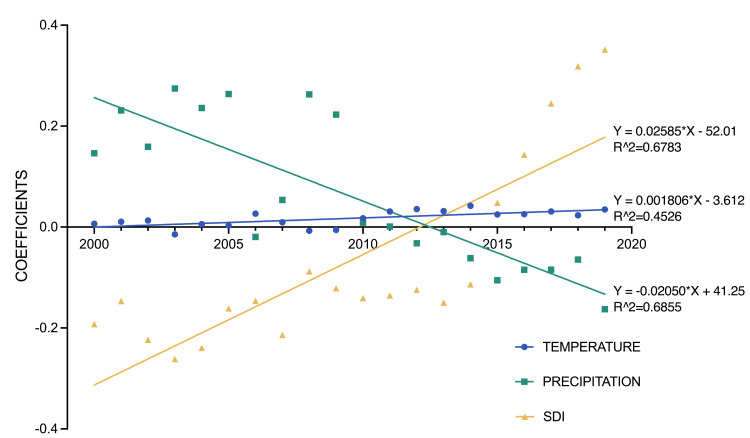
Association of malaria ASIR with annual average temperature and precipitation, and SDI, by year. ASIR – age-standardised incidence rate, SDI – socio-demographic index.

In subgroup analyses of different years of multivariable quasi-Poisson GLM regression models, we found that the impact of the national annual average temperature on malaria incidence increased over time: the coefficients of AT increased from 0.0067 (95% CI = 0.0062, 0.0071) in 2000 to 0.0348 (95% CI = 0.0342, 0.0354) in 2019, indicating that with a 1°C increase in national annual average temperature, malaria ASIR would increase by 0.67% (95% CI = 0.62, 0.72) in 2000 and 3.54% (95% CI = 3.48, 3.60) in 2019% (*P*-value <0.05). The impact of SDI on malaria incidence also increased over time. Before 2015, when SDI increased, the ASIR of malaria would decrease; however, from 2015 to 2019, a 0.01 increase in SDI meant that the ASIR of malaria would increase by 0.48% to approximately 3.58% (*P*-value <0.05) (**Figure 5**).

## DISCUSSION

To the best of our knowledge, this is the first study to estimate the association of annual average temperature and precipitation with malaria incidence on a global scale from 2000 to 2019, thus providing a comprehensive overview of the global distribution of national annual average temperature and precipitation, and their association with malaria incidence. We found that malaria was still impacting people’s health and livelihoods in malaria-endemic countries, with 231.4 million malaria cases occurring worldwide in 2019. National annual average temperature and precipitation were positively associated with malaria incidence, with an increase in ASIR of 2.01% (95% CI = 2.00, 2.02) following a 1°C increase of national annual average temperature, and an increase in ASIR of 6.04% (95% CI = 6.00, 6.09) following a one mm increase of national annual average precipitation per day. In the subgroup analysis, we found that malaria incidence in Asian countries was most affected by temperature, while the incidence in African countries was most affected by precipitation. In different age groups, children <5 years of age were most affected by both temperature and precipitation. We additionally found that the impact of the national annual average temperature on malaria incidence increased over time. Our findings could add to previous studies and help with understanding the impact of climate factors on malaria transmission, identifying vulnerable regions, and preparing for potential changes in disease dynamics associated with broader climatic shifts, thus informing malaria prevention and control efforts amidst climate change to advance movement towards the global goal of malaria elimination.

There were 252.9 million malaria cases worldwide in 2000, with a global ASIR of 3946.18 per 100 000 population. Notably, although the total number of malaria cases increased to 263.7 million in 2010, there was a simultaneous decrease in the ASIR to 3877.86 per 100 000 population. This observed decrease in ASIR may be attributed to multiple factors, including population growth, changes in diagnostic criteria, and variations in reporting practices. The global population growth during this period might have outpaced the increase in malaria cases, contributing to the decline in ASIR. Besides the gold standard for diagnosing malaria – microscopy of thick and thin blood films [24], other methods include malaria rapid diagnostic tests [25], parasite lactate dehydrogenase (pLDH) rapid diagnostic tests [24], and nucleic acid testing [26]. As diagnostic criteria and technologies have evolved over time, they have impacted the reporting of malaria cases, which in turn might have led to more accurate and widespread detection of malaria cases, consequently influencing the total reported case numbers. Variations in reporting practices among different countries and regions also play a role in the accuracy and completeness of malaria case reporting [27]. It is important to acknowledge these influencing factors when interpreting the trends in ASIR over time.

We examined national annual average temperature and precipitation changes across diverse countries from 2000 to 2019. Our study characterises country-level variations without explicitly attributing them to global climate change. The notable temperature increase in countries like Nigeria, Nepal, DR Congo, and Ecuador reflects regional climatic shifts, aligning with global climate change expectations. However, caution is needed in attributing these shifts solely to global climate change, as local factors like land use, urbanization, and atmospheric circulation patterns may contribute [28–31]. Similarly, observed precipitation changes in Tuvalu, China, and South Korea signify localised variations, impacting vector habitats and malaria transmission [12,14]. While trends align with global climate change expectations, distinguishing global contributions from regional influences requires further investigation. Our findings contribute to understanding climate-malaria associations, recognising the intricate relationship between local and global climatic factors.

We found a positive dose-response relationship between national annual average temperature and the ASIR of malaria. Specifically, a 1°C increase in temperature was associated with a 2.01% increase in ASIR. Notably, subgroup analyses demonstrated varying impacts across regions, with Asian countries exhibiting the highest sensitivity (22.69% increase) and African countries the lowest (1.35% increase). *Anopheles* mosquitoes, the transmission vector of malaria, are poikilotherms with life history characteristics strongly dependent on the ambient temperature, including the length of the gonotrophic cycle, fecundity, biting rate, longevity, and development of immature mosquitoes [32]. Previous studies observed that increasing temperature reduced the development time of the immature stages of *Anopheles* larvae: at 25 C, *Anopheles gambiae sensu stricto* mean time to eclosion = 11.9 days (95% CI = 11.6, 12.1), and *Anopheles arabiensis* = 12.6 days (95% CI = 12.4, 12.8); at 30°C, *Anopheles gambiae sensu stricto* = 9.6 days (95% CI = 9.4, 9.9), and *Anopheles arabiensis* = 10.5 days (95% CI = 10.3, 10.8) [33,34]. Increasing temperature also decreased the time to pupation of *Anopheles* larvae [35] and decreased the time to hatching, without reducing the hatching rate of *Anopheles* eggs [36]. Moreover, studies also found that the efficacy of insecticides reduced with increasing temperature [37,38]. However, extremely high temperatures could reduce the transmission of malaria, with the optimal malaria transmission temperature being predicted at 25°C [39]. In countries with high annual average temperatures, such as Ghana and Nigeria, an inverse temperature-incidence rate relationship was observed [13], which partly explained our finding that African countries were the least affected by temperature.

Moreover, we found that the impact of the national annual average temperature on malaria incidence increased over time, as indicated by the rising coefficients of AT from 0.0067 in 2000 to 0.0348 in 2019. This suggests an escalating effect, with a 1°C increase in temperature associated with a 0.67% increase in ASIR in 2000 and a 3.54% increase in 2019. This trend aligns with predictions about the impact of global warming on vector-borne diseases, suggesting an intensified transmission of malaria associated with temperature increases [40–42]. Human activities had already warmed the planet by 1°C above pre-industrial levels. The 2015 Paris Agreement signed by 195 countries limited global warming below 2°C, while some researchers declared global warming must stay below 1.5°C [43]. Although the increase in the incidence rate of malaria did not seem to be too serious compared with extreme weather events, rising sea levels, destruction of coral reefs, loss of biodiversity, ocean acidification and deoxygenation, and extreme heat, efforts are still needed to control malaria amidst the global warming [44].

We observed a positive dose-response relationship between national annual average precipitation and malaria ASIR. An increase of one mm per day was associated with a 6.04% increase in ASIR. Malaria transmission is mainly seasonal, with rainfall playing a strong role in causing an outbreak [45,46]. A previous study in India found that the rainfall cutoff required for a malaria outbreak ranged from >70 to >600 mm, and the outbreaks occurred with minimum rainfall cutoff in arid regions compared to other places [47]. Heavy rainfall would probably destroy both larvae and, in some instances, adult mosquitoes, which was consistent with our finding that in Asian countries, when national annual average precipitation increased by one mm per day, the ASIR of malaria would decrease by 8.38%. However, under certain conditions, heavy rainfall with long duration could promote the transmission of malaria. A study from Sri Lanka reported the ability of *Anopheles culicifacies* to tolerate salinity changes during the rainy season [48]. Besides the tolerance of salinity changes, heavy rainfall with long duration could also provide plenty of breeding sites, which can eventually cause an outbreak.

A worrying finding of our study was that children <5 years of age were most affected by both temperature and precipitation, with an increase in the malaria incidence rate of 2.76% (95% CI = 2.75, 2.77) and 8.07% (95% CI = 8.04, 8.09), respectively, following a one unit increase of national annual average temperature and precipitation. Children were the most vulnerable group affected by malaria, and children <5 years of age had the highest malaria incidence rate among all age groups [4]. While young children bore the main burden of malaria in high transmission areas [49], they were also facing other health problems, such as anaemia, nutritional deficiencies, genetic conditions, and other parasitic, viral, and bacterial infections [4]. Therefore, targeted interventions for young children to prevent malaria are required, especially in malaria-endemic countries.

We also found that a country-level SDI increase of 0.01 resulted in a ASIR decrease of 0.78% (95% CI = 0.77, 0.79). As a composite, a country with an SDI of 0 would have a theoretical minimum level of development relevant to health, while a country with an SDI of 1 would have a theoretical maximum level [21]. Importantly, the WHO’s road map for neglected tropical diseases 2021–2030 adopted the ‘Leave No One Behind’ agenda, which comprises taking steps to end extreme poverty, curb inequalities, confront discrimination, and fast-track the progress of countries the most undeveloped countries [50]. Therefore, immediate and effective actions need to be taken to establish an international cooperating strategy to reduce the burden of malaria in low SDI countries. However, in North, South, and Central American regions, the ASIR of malaria would increase by 11.63% and 3.05%, respectively, when SDI increased by 0.01. Furthermore, the influence of SDI on malaria incidence also increased over time. Initially, before 2015, increasing SDI correlated with a decrease in ASIR, reflecting socioeconomic improvements and successful malaria control efforts. However, from 2015 to 2019, a 0.01 increase in SDI led to a 0.48% to 3.58% increase in ASIR, suggesting a possible plateau in the effectiveness of existing interventions and the emergence of new challenges. To address this, comprehensive strategies should include employing adaptable control measures, strengthening health systems, conducting community engagement, continuing ongoing research, and establishing international collaboration to address the global malaria challenge.

This study had some limitations. First, this was a worldwide observational study encompassing all the malaria-endemic countries with meteorological observation data, which was representative and statistically efficacious; however, caution is needed when making causal inferences, since not all the malaria-endemic countries had meteorological observation data. Additionally, this was an ecological study based on linked aggregated data on malaria incidence and meteorological data, which might have caused ecological fallacies. Despite regional variations, the consistency in patterns is noteworthy. Temperature consistently showed a positive association, while the effect of precipitation varied by region. SDI demonstrated diverse effects, emphasising the importance of considering sociodemographic factors in malaria incidence. Our findings could inform further studies based on individual data and efforts in controlling malaria in the context of global climate change. Second, there are inherent limitations of using the GBD database: only yearly and nation-level data were available, and when the original data were sparse or missing, we have had to estimate the burden of disease through the models. Third, we did not analyse the lag time pattern in our study as we used annual data, which generally cannot be used for this purpose.

## CONCLUSIONS

Our study helps clarify the relationships between SDI, climate factors, and malaria incidence and highlights a notable temporal shift in these dynamics. The heightened impact of temperature and precipitation on malaria incidence in children <5 years of age underscores the urgency of addressing climate-related influences. In response to these findings, we advocate for a multifaceted approach to malaria prevention and control, incorporating innovative technologies, fostering cross-sectoral collaborations, and recognising the increasingly critical role of adaptive measures in malaria control strategies, particularly those tailored to climate vulnerabilities. International collaboration and resource mobilisation remain key to shared learning and collective action. Continued investment in research and development, alongside exploring novel tools and vaccines, will be crucial for facing and resolving evolving challenges. A comprehensive global collaboration framework is needed for facilitating coordinated efforts, enabling resource sharing, and establishing joint initiatives, with an added emphasis on climate-responsive strategies. These strategic enhancements could advance global malaria control efforts toward the goal of elimination.
